# Effect of nanoparticle density on the kinetics of SPP-assisted plasmonic assembly

**DOI:** 10.1038/s41598-025-14058-0

**Published:** 2025-08-05

**Authors:** Kethahalli Shivappa Mahadeva Prasad, Rajeev K. Sinha, Aswini Kumar Mohapatra, Aseefhali Bankapur

**Affiliations:** 1 Manipal Institute of Applied Physics, Manipal Academy of Higher Education, Manipal, 576104 India; 2 Department of Physics, Birla Institute of Technology, Mesra, Ranchi, 835215 India; 3 Department of Respiratory Medicine, Kasturba Medical College, Manipal Academy of Higher Education, Manipal, 576104 India

**Keywords:** Plasmonic nanoparticle assembly, Nanoparticle density, Plasmonic assembly size, Solution-based SERS, Industry, innovation and infrastructure, Techniques and instrumentation, Optical manipulation and tweezers, Nanophotonics and plasmonics, Raman spectroscopy

## Abstract

The dynamic assembly of plasmonic metal nanoparticles (PMNPs) in an aqueous medium as a Surface-enhanced Raman spectroscopy (SERS) substrate offers advantages for analyzing liquid samples, as it generates 3-dimensional intraparticle hotspots. The surface plasmon polariton (SPP) assisted surfactant-free reversible assembly of plasmonic nanoparticles (NPs) is one of the latest methods, and it stands as a promising approach for conducting SERS measurements on molecules that demand a physiological environment. However, the assembly process is dynamic and requires a thorough analysis of the behavior of NPs in the combined forces of fluid convection and plasmonics. This study investigates the kinetics of the plasmonic assembly of gold nanoparticles (AuNPs) and the influence of NP density through microscopy and SERS monitoring over 60 min. The study reveals that the assembly size and density grow gradually at an NP density-dependent rate. The SERS intensity of the analyte molecules increases rapidly and begins to decrease gradually after reaching a maximum value at an assembly time that is NP density-dependent. The assembly time at maximum SERS intensity is found to be inversely proportional to the NP density. Notably, when SERS intensity is maximum, the assembly radius remains constant at 119±1 μm for all the NP densities.

**Supplementary Information:**

The online version contains supplementary material available at 10.1038/s41598-025-14058-0.

## Introduction

Surface-enhanced Raman spectroscopy is a reliable method to enhance the Raman signal intensity and has proven its potential in detecting analytes at attomolar concentrations^1^. In SERS, signal enhancement arises primarily from two mechanisms: electromagnetic and chemical. The dominant electromagnetic mechanism is based on the excitation of localized surface plasmon resonances (LSPR) in metallic nanostructures when illuminated by light. This leads to the generation of intense localized electromagnetic fields, especially at regions known as “hotspots,” such as nanogaps or sharp edges of NPs. When a molecule is present in these hotspots, the Raman signal is amplified significantly^2^. The secondary mechanism, known as chemical enhancement, involves charge transfer between the metal surface and the adsorbed molecule. This interaction modifies the molecule’s polarizability or introduces new electronic states, enhancing specific vibrational modes. Therefore, the performance of a SERS substrate critically depends on its design, including material composition, surface morphology, and nanostructural features, all of which influence its ability to detect trace-level analytes with high sensitivity. Over the past few decades, the scientific community has been actively involved in developing and optimizing SERS substrates to create robust and versatile platforms capable of supporting a wide range of applications with minimal operational constraints, such as stability, shelf life, analyte accessibility, surface adsorption, background interference, environmental sensitivity, cost, and reproducibility^3,4^. During this period, various SERS substrates have evolved through multiple advancements in material design and nanofabrication techniques. Initially, roughened silver electrodes were used^5^, followed by the introduction of silver and gold (Au) colloids^6,7^. Researchers have explored a range of SERS substrates, including metal films with varied thicknesses and nanostructuring^8–10^. Subsequently, hybrid fabrication strategies that integrate colloidal NPs with nanostructured metal films have been developed, offering improved enhancement and stability^11,12^. Across the wide array of SERS substrates developed, colloidal solutions of PMNPs are preferable for SERS measurements due to their easily tunable optical, chemical, and physical properties, which can be tuned to match the wavelength of Raman excitation for optimal signal enhancement. Besides, the dynamic and reversible assembly of PMNPs in aqueous environments enabled sensitive biomolecule and biomarker detection by facilitating the formation of dense interparticle nanogaps, which act as electromagnetic hotspots that amplify Raman signals. Such PMNP assemblies have been achieved either using thermophoresis^13,14^ or SPPs^15,16^. Lin et al.^13^ demonstrated a reversible and dynamic plasmonic NPs assembly using light-directed plasmon-enhanced thermophoresis. Recently, Zheng et al.^14^ developed a flexible and reusable SERS detection strategy by integrating an Au-coated optical fiber with a microfluidic chip. This system enabled the reversible gold nano bipyramids (AuNBPs) accumulation through thermophoresis, achieving SERS detection limits as low as 10⁻¹⁰ M for RhG solutions. However, both approaches relied on the use of high laser power and surfactants, which limit their compatibility with biological systems due to potential risks such as cellular damage, protein denaturation, or disrupted biomolecular functionality^17^. Approximately ten years ago, Patra et al.^15^ formed the reversible assembly of PMNP aggregates via SPP excitation on a metal film using the Kretschmann configuration and demonstrated single-molecule SERS detection in an aqueous medium. However, the study used a 532 nm excitation wavelength and assembled micron-sized silver NP aggregates, hindering its application for biological systems. Ghanashyam et al.^16^ recently extended SPP-based PMNP assembly to bio-friendly near-infrared (NIR) excitation and AuNPs. The study demonstrated interparticle nanogaps tuning, acquired a reproducible SERS signal for SR101 dye up to 10⁻¹⁰ M concentration, and detected low concentration dsDNA with a reduced power density of 100 nW µm⁻².

Although previous studies on SERS using reversible assembly of PMNPs as substrates have examined the influence of factors such as excitation power density, interparticle nanogaps, and analyte concentration on the resulting SERS intensity. Most earlier studies did not give much attention to how the concentration of NPs in the solution impacts the assembly kinetics or dynamic aspects of nanoparticle assembly, including the rate at which nanoparticles accumulate at the assembly site, the growth in the assembly size, nanogap formation dynamics, etc., and its effect on SERS. Notably, earlier reports often involved the dilution of colloidal NP solutions before initiating plasmonic assembly. This dilution was primarily aimed at slowing down the assembly process, thereby making it easier to arrest the assembly at a desirable stage and obtain reproducible SERS spectra. However, insights from static SERS substrates such as NP films or nanostructure arrays have established that the density of plasmonic NPs plays a crucial role in determining the magnitude of SERS enhancement, as demonstrated in several studies^18–21^. By analogy, it becomes important to understand whether and how NP density influences the dynamics of PMNP assembly under optical excitation in solution-phase systems.

To address this gap, the present study systematically investigates the effect of NP density in colloidal suspension on the kinetics of dynamic plasmonic assembly. This is achieved by simultaneously monitoring changes in SERS signal intensity and capturing the corresponding microscopic assembly events, thereby providing a comprehensive understanding of the correlation between NP concentration, assembly behavior, and SERS performance.

## Experimental

### Material

Gold (III) chloride trihydrate [HAuCl_4_] (≥ 99.99%), L-ascorbic acid (≥ 99%), Chitosan (mol wt: 50 − 190 kDa, viscosity: 20–300 cP), Acetic acid Glacial (≥ 99.0%), and Sulforhodamine 101 (SR101), were obtained from Sigma-Aldrich, and Ultra-pure water (ρ > 16.5 MΩ) was used throughout the experiments.

### Synthesis of AuNP colloidal solution

Chitosan-capped gold nanoflowers (Chi-AuNFs) were synthesized following Nhung et al.‘s protocol^22^. A 0.05 wt% chitosan solution was prepared in 1 v/v% acetic acid. To 3 mL of this solution, 70 µL of 25.3 mM HAuCl₄ was added under magnetic stirring for 30 min. Subsequently, 150 µL of 1% L-ascorbic acid was added, and stirring continued for another 30 min. The change in the color of the solution from colorless to blue confirmed the formation of AuNFs.

### Preparation of SR101 solutions containing different NP densities

Five different NP density solutions were prepared by varying the volume percent of the as-prepared NP solution and DI water. A 10 µM SR101 dye solution was used as a standard sample for the SERS experiments. Five solutions (A, B, C, D, and E) of 10 µM SR101 were prepared with varying NP densities of 7%, 14%, 21%, 28%, and 35%, respectively, by adjusting the volume ratios of 100 µM SR101 stock solution, as-prepared AuNP colloidal solution, and DI water.

### Instrumentation

**Fig. 1:**
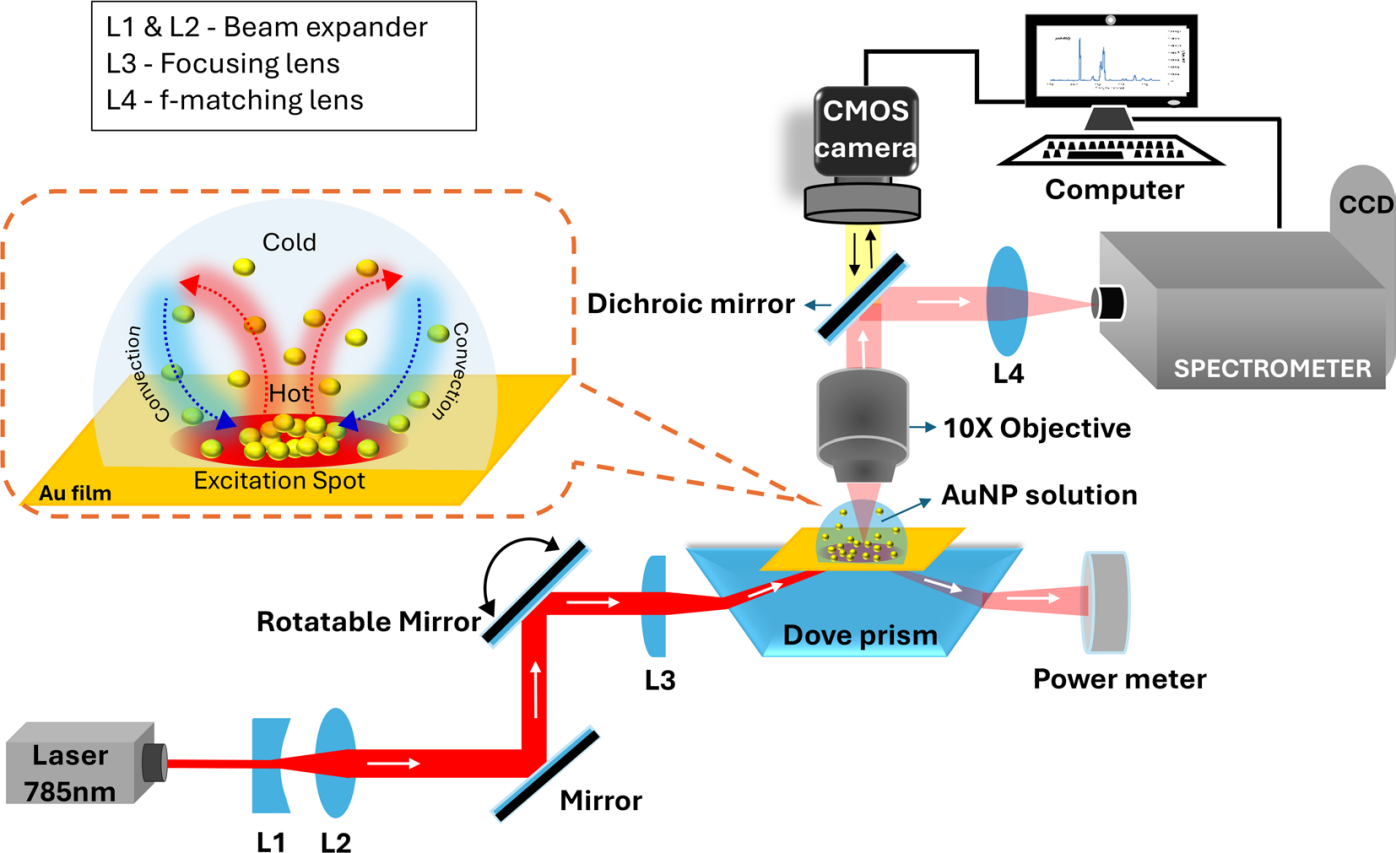
The schematic diagram of the optical setup for plasmonic assembly of AuNPs and SERS measurement. The inset shows the mechanism of plasmonic assembly under far-field convection forces and near-field plasmonic forces.

The dynamic assembly of plasmonic NPs and SERS spectral acquisition were conducted using a custom-built optical setup, illustrated schematically in Fig. 1. A 785 nm diode laser (Ondax, USA) was the excitation source. A beam expander was positioned adjacent to the laser to achieve the desired beam size. The laser beam was elevated to the level of a dove prism (NBK-7) using a periscopic arrangement and focused onto its top facet. Because of its trapezoidal geometry, the dove prism helps in the easy implementation of the Kretschmann configuration, enabling simultaneous video or image capturing and SERS signal acquisition. A borosilicate coverslip with a 50 ± 5 nm thick Au coating, prepared using a vacuum thermal unit (Hind High Vacuum, India), was adhered to the top facet of the dove prism with refractive index matching oil. SPPs were generated by placing the AuNPs colloidal solution on top of the Au film, and the optimal SPPs were achieved by tuning the incident angle using a rotatable mirror. An optical detector was positioned on the opposite side of the Dove prism to monitor the coupling power. The assembly process of the NPs was captured through imaging and video recording using a CMOS camera (DSFi3, Nikon, Japan). A 10X microscopic objective (Plan Fluor, W.D = 16 mm, N.A. = 0.3) was employed for imaging and SERS signal collection. A f-matching planoconvex lens couples the collected signals to a spectrometer (iHR 320, Horiba, Japan). The spectrometer is configured with a 1200 gr/mm holographic grating and a liquid-nitrogen cooled charge-coupled device. All spectra were acquired with a laser power of 7 mW, corresponding to a power density of 0.3 µWµm^− 2^ for a focal spot of 170 μm diameter and a fixed acquisition time of 1 s per individual spectrum. Even though the NP assembly and SERS measurements can be achieved at laser powers as low as 10 µW (power density ~ 100 nWµm^− 2^), we have used 7 mW in order to maintain a good signal-to-noise ratio across all the measurements. These power density levels are far lower than those used in SERS measurements and reported to cause no/minimal photothermal damage to the analytes^23,24^.

In the SPP-assisted plasmonic assembly approach, AuNPs are driven to the SPP excitation spot on the Au film by the far-field fluid convection forces generated by the thermal gradients and held in place by the near-field electric gradient forces generated by the SPPs, as shown in the inset of Fig. 1. On SPP excitation, the Au thin film heats up and creates a thermal gradient in the AuNP solution, which sets up convection in the solvent (water), circulating NPs between hot and cold regions. Later, the circulating NPs, while passing through the hot SPP excitation region, experience plasmonic gradient forces set up there and get trapped.

### AuNP characterization

**Fig. 2:**
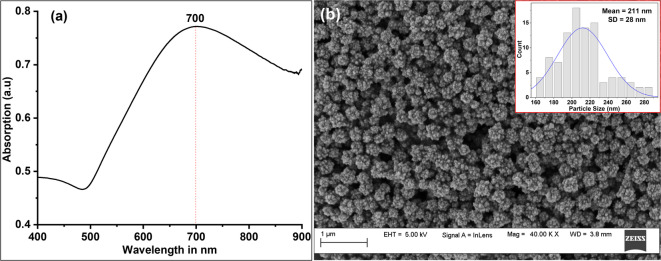
The characterization of prepared Chi-AuNFs: (**a**) The UV-vis absorption spectra, (**b**) FE-SEM image. The inset shows the particle size histogram extracted from the image.

The absorption spectra of the synthesized Chi-AuNFs were recorded using a UV/Vis spectrophotometer (V-650, Jasco, Japan). Analyzing the peak position and width of UV/Vis absorption spectra of NPs provides insight into NP size and monodispersity^25^. Fig. 2(a) presents the absorption spectrum of the prepared Chi-AuNFs, with the LSPR peak positioned at 700 nm, closely aligning with the 785 nm excitation wavelength. For SERS measurements, the LSPR peak of AuNPs must be near the excitation wavelength. The spectrum exhibits a broad peak, indicative of the polydispersity typically associated with branched or structured NPs^26^. The precise size and morphology of the prepared NPs were determined using field emission scanning electron microscopy (FE-SEM) (Carl ZEISS Sigma, Germany) imaging. Fig. 2(b) displays an FE-SEM image of the AuNFs at 40k magnification with a particle size histogram as an inset, revealing an average size of 211±28 nm with a characteristic flower-like morphology. Such structured NPs are preferred for SERS applications, as their sharp edges and corners promote the formation of numerous hotspots, leading to enhanced SERS signal amplification^27 ^especially when they are assembled with a favorable interparticle nanogap. Zeta potential and dynamic light scattering (DLS) measurements were conducted with a nanoparticle analyzer (SZ-100, Horiba, Japan). The observed UV/Vis absorption peak, size, and zeta potential of the prepared chi-AuNFs align well with literature reports^16,22 ^making them suitable for SPP-assisted plasmonic assembly experiments.

## Results and discussion

**Fig. 3:**
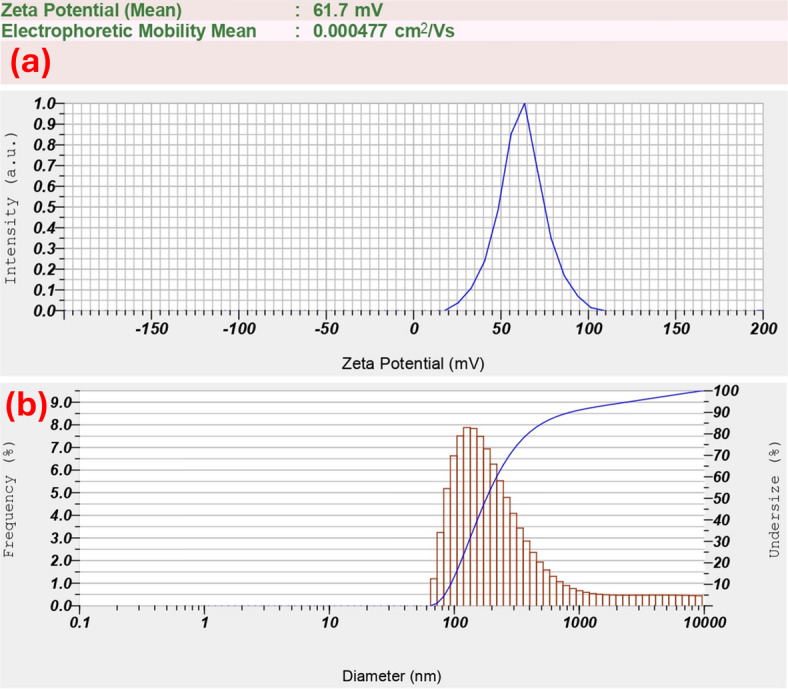
(**a**) The zeta potential graph and (**b**) size distribution histogram of Chi-AuNFs.

**Fig. 4:**
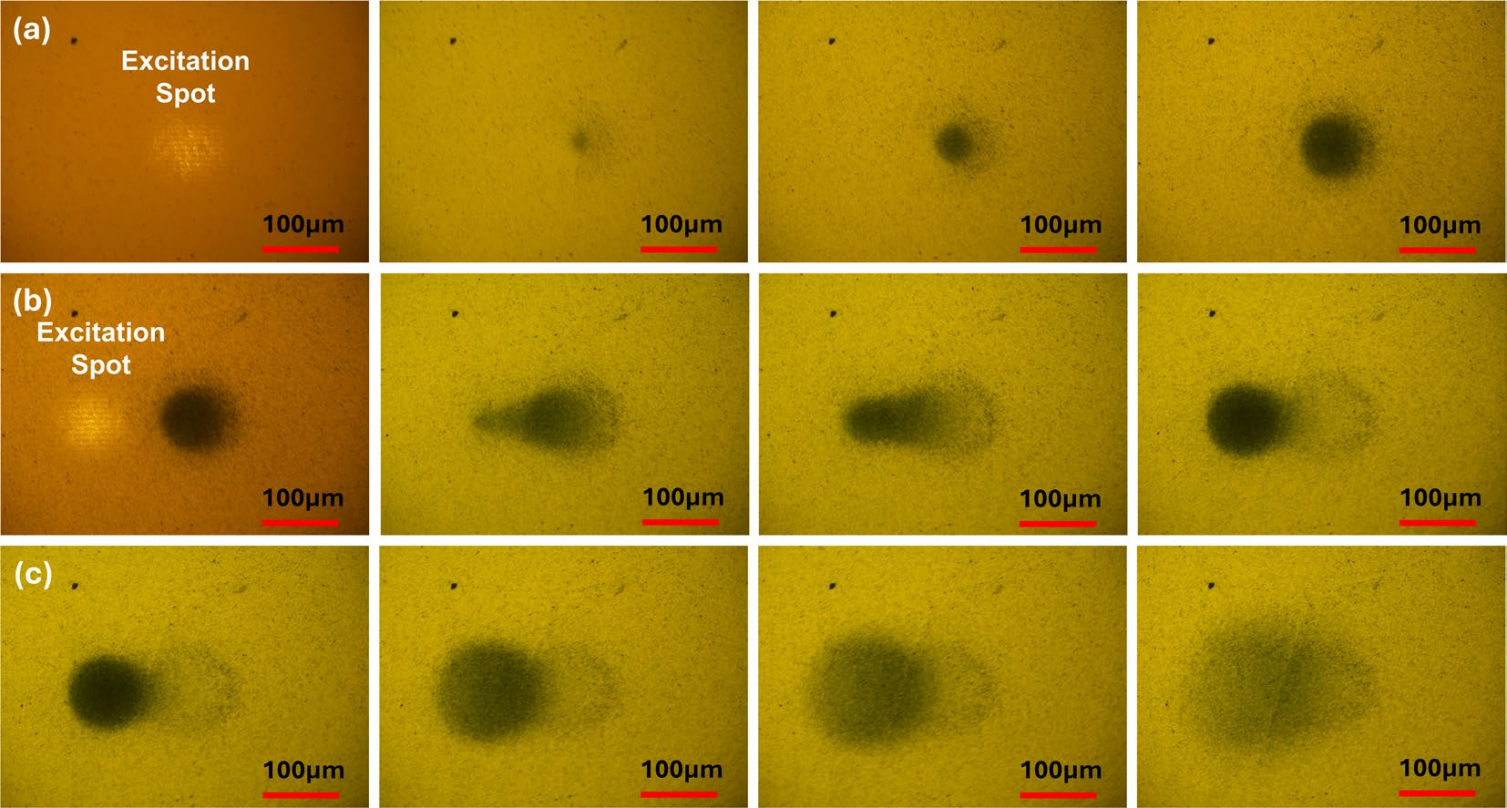
Image sequence showing (**a**) the plasmonic assembly of as-prepared Chi-AuNFs, (**b**) the transport of assembled AuNFs by shifting the laser spot, and (**c**) the redispersion of as-prepared Chi-AuNFs on removing the laser excitation.

In our previous study^16^we successfully achieved SPP-assisted dynamic and reversible assembly of chi-AuNFs with a positive surface charge and an average size of 125 nm. In contrast, the NPs with negative surface charge and sizes lower than 50 nm did not show plasmonic assembly, indicating the influence of either surface charge or size, and both on SPP-assisted plasmonic assembly. Therefore, it is essential to characterize the surface charge, size, and assembly behavior of the synthesized chi-AuNFs before evaluating the effect of NP density on assembly kinetics. Zeta potential measurements confirmed a stable positive surface charge with a zeta potential of + 61.7 mV, and DLS measurements found an average particle size of 180.8 nm, z-average of 197.5 nm, and polydispersity index of 0.479. Fig. 3(a) and Fig. 3(b) present the zeta potential graph and the corresponding size distribution histogram of the chi-AuNFs. The assembly behavior of AuNPs under the SPP field was examined by subjecting them to the plasmonic assembly under a coupling power of 7 mW. The NP assembly was observed within a few minutes, and a dense assembly was formed in 60 min. Fig. 4(a) presents sequential images depicting the plasmonic assembly process of the Chi-AuNFs. Fig. 4(b) shows the assembly transport, and Fig. 4(c) illustrates the redispersion of the assembly upon switching off the laser, confirming its reversible nature (a supplementary video shows all these processes).

**Fig. 5:**
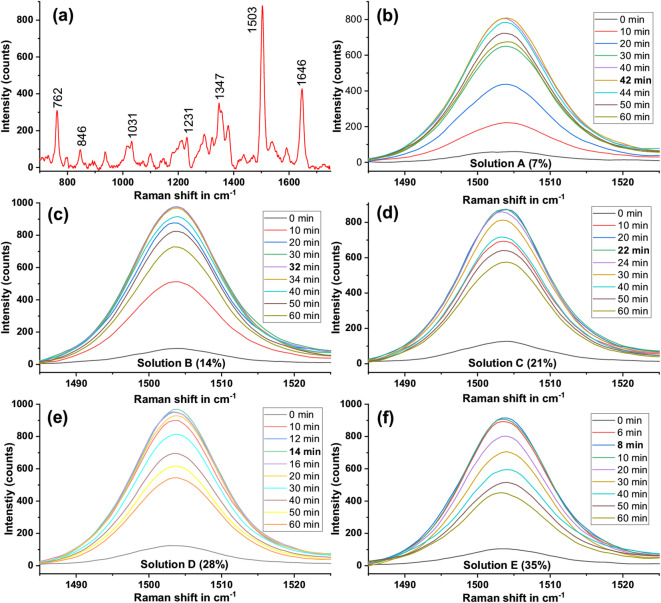
(**a**) A typical SERS spectrum of 10µM SR101 dye and SERS intensity variation with growing NP assembly for different NP densities (**b**) 7%, (**c**) 14%, (**d**) 21%, (**e**) 28%, and (**f**) 35%. Each spectrum is the average of three measurements.

The NP assembly formed within the aqueous medium can be quickly transported to various locations on the Au film by shifting the excitation spot from place to place, effectively serving as a dynamic transportable SERS substrate^16^. However, as the assembly process is continuous, the assembly density (AuNPs per unit volume of the assembly) and time required to reach the saturation point (time at which assembly density stabilizes) depend on factors like NP density in the prepared colloidal solution and laser powers used. Hence, it is important to investigate the dependence of NP assembly kinetics on NP density and its influence on SERS signals from the analytes to time the signal acquisition. To examine the influence of NP density on assembly density and the resultant SERS signal, we used 10 µM SR101 dye containing different NP densities (solutions A-E) as a standard sample. Fig. 5(a) presents the typical SERS spectrum of SR101 dye, recorded with a coupling power of 7 mW and an acquisition time of 1 s. The Raman peaks of SR101 were observed at 762, 846, 1031, 1231, 1347, 1503, and 1646 cm^− 1^, with most of these peaks aligning well with previously reported values in the literature^16^.

To monitor SERS intensity variation with the assembly time for different NP densities and further analysis, we considered the intensity of the 1503 cm⁻¹ spectral peak of SR101. The experiment was performed by subjecting all the prepared SR101 solutions with varying NP densities (solutions A-E) individually to the plasmonic assembly under a coupling power of 7 mW, and the corresponding SERS spectra were recorded. To examine the variation in SERS intensity with growing assembly, spectra were recorded at 2-minute intervals, and the corresponding NP assembly images were simultaneously captured for up to 60 min. The assembly experiments and related temporal SERS measurements were performed in triplicate to demonstrate the reproducibility of the events and results. Fig. 5 (b-f) presents the overlaid plots of the 1503 cm⁻¹ SERS peak of SR101 recorded at different time intervals of the assembly process for NP densities ranging from 7 to 35% (solutions A-E), respectively. A key observation is that, across all the solutions, the SERS intensity rapidly increases with the growing assembly and reaches a maximum value at different times for solutions A-E, then gradually diminishes with further growth of the NP assembly.

**Scheme 1:**
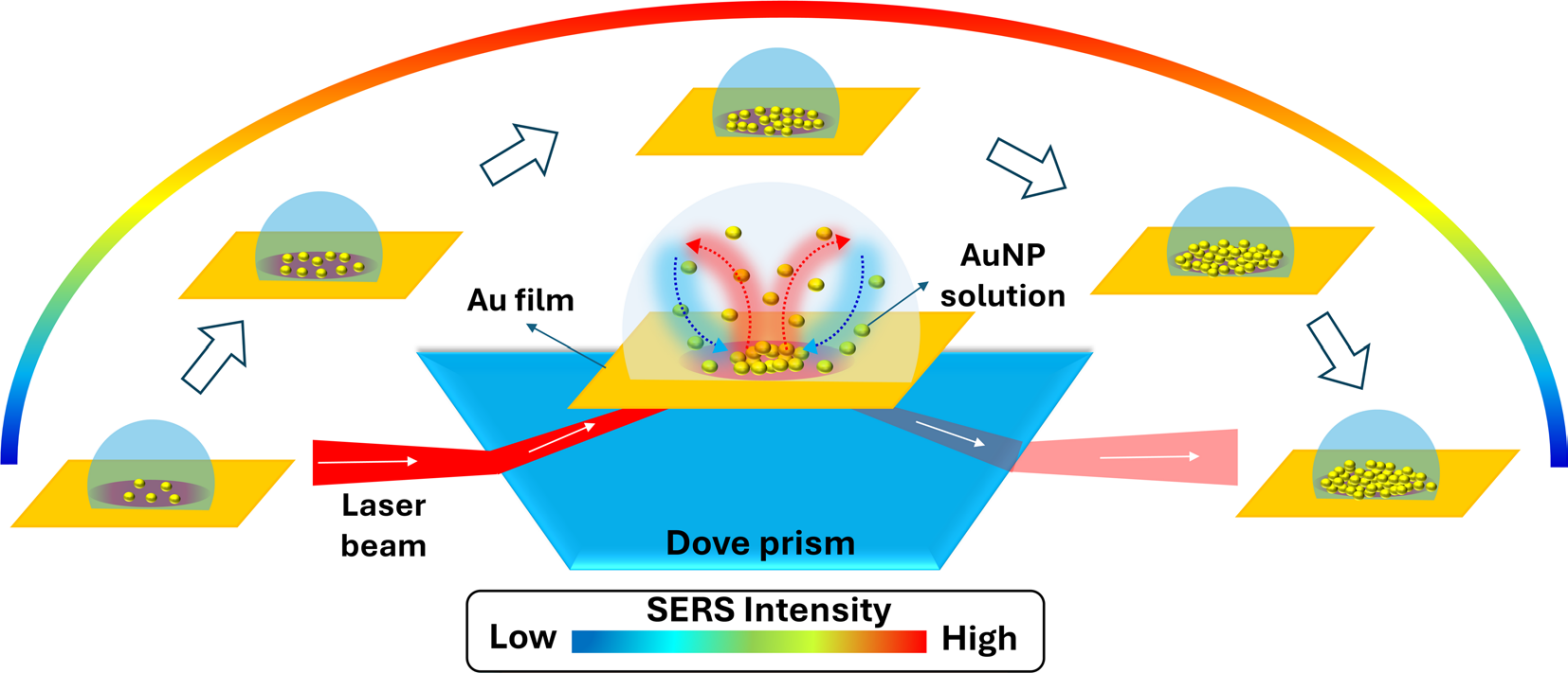
Pictorial representation of the proposed mechanism of assembly kinetics and related SERS intensity variation.

The possible reasons for this kinetic behavior can be explained as follows: (i) Firstly, on SPP excitation, the thermal gradient sets up convection in the solvent (water) which circulates NPs between hot and cold regions, (ii) NPs while passing through the hot SPP excitation region get trapped due to the local plasmonic gradient forces, (iii) as time passes more and more NPs from the colloidal solution accumulate at the excitation spot and assembly density increases, (iv) initially when assembly density is less, interparticle nanogaps are wider and few analyte molecules are excited resulting in lower SERS intensity, (v) as the assembly density grows, the interparticle nanogap decreases, and more analyte molecules are excited, leading increase in SERS intensity, and (vi) after a given time, the density grows to a level that the interparticle nanogap reduces below an optimum value and also the dense packing of NPs in z-direction block SERS signals from reaching the collection optics leading to fall in the detected SERS intensity. The proposed mechanism of assembly kinetics and related SERS intensity variation is pictorially presented in Scheme 1.

**Fig. 6:**
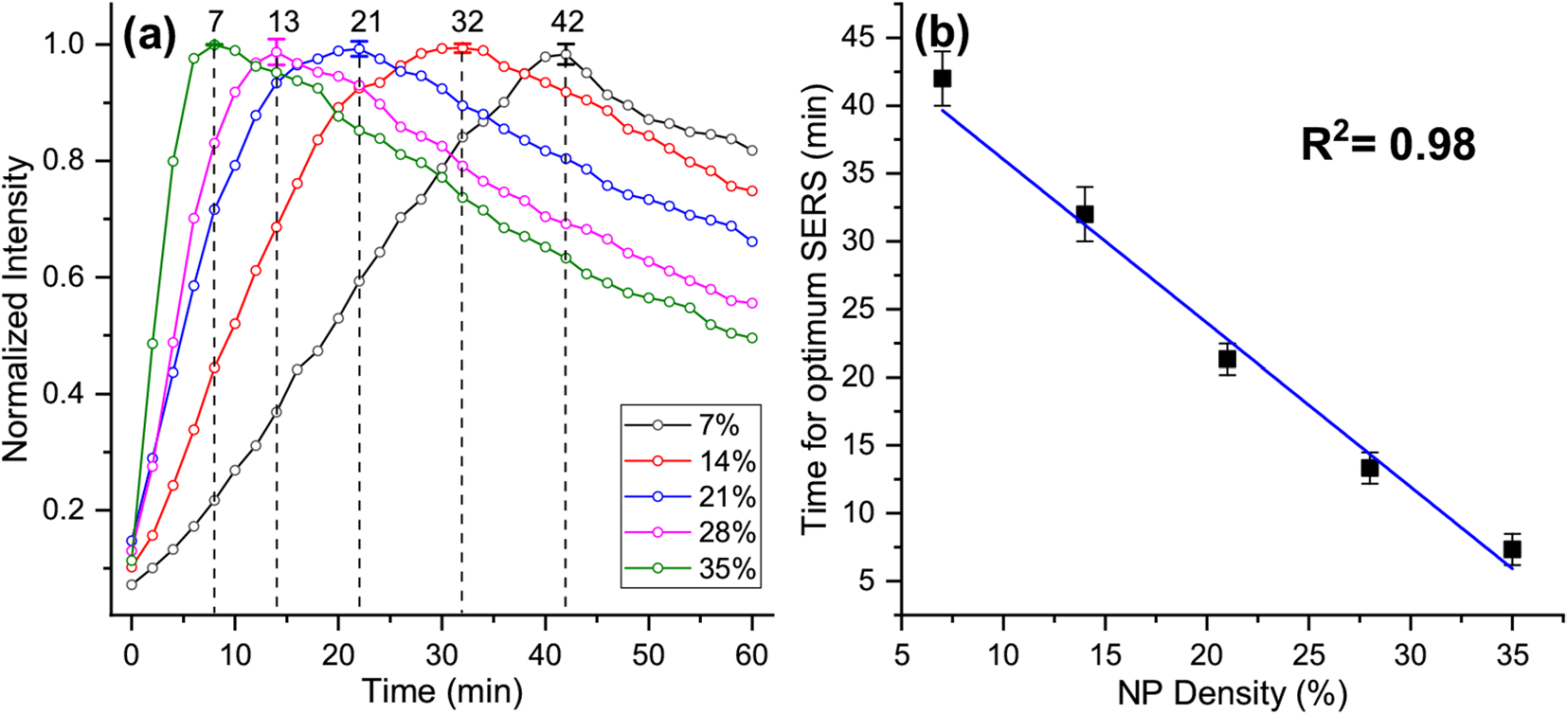
(**b**) The plot for SERS measurement time versus the corresponding intensity counts of the peak at 1503 cm⁻¹, (**b**) The plot for NP density versus time taken to attain optimum SERS signal. The data is the average of three measurements.

Fig. 6(a) compares the plots of SERS intensity variation over the assembly time for the solutions A-E. From the graph, the time taken to attain the optimal SERS signal was found to vary depending on the NP density in the solution, with a shorter time for higher NP density and a longer time for lower NP density. This is because, for a given thermal gradient, the fluid convection-induced NP circulation rate is NP density-dependent, leading to the early observation of optimum SERS conditions in higher NP density solutions. Conversely, the lower NP density in the solution necessitates a longer accumulation time at the excitation spot to generate an optimal SERS condition. In our previous report^16 ^we have experimentally proved that the SERS intensity is interparticle nanogap dependent; in plasmonic assembly, it is best for the nanogaps created with 7 mW excitation power for a given NP density. In that experiment, the NP density was very low (5:1000 dilution of prepared AuNPs), and SERS measurements were performed after assembly saturation was achieved to remove the influence of varying assembly density on SERS intensity. In this study, we observed that even for the optimum excitation of 7 mW, the SERS intensity is assembly time-dependent if NP densities are relatively closer (~ 1:3 for 35% to – 1:14 for 7%) to the prepared NP density. To illustrate the relationship between NP density and the time taken to attain the optimum SERS signal, a plot was generated, as shown in Fig. 6(b), revealing a linear trend with an inverse proportionality.

**Fig. 7:**
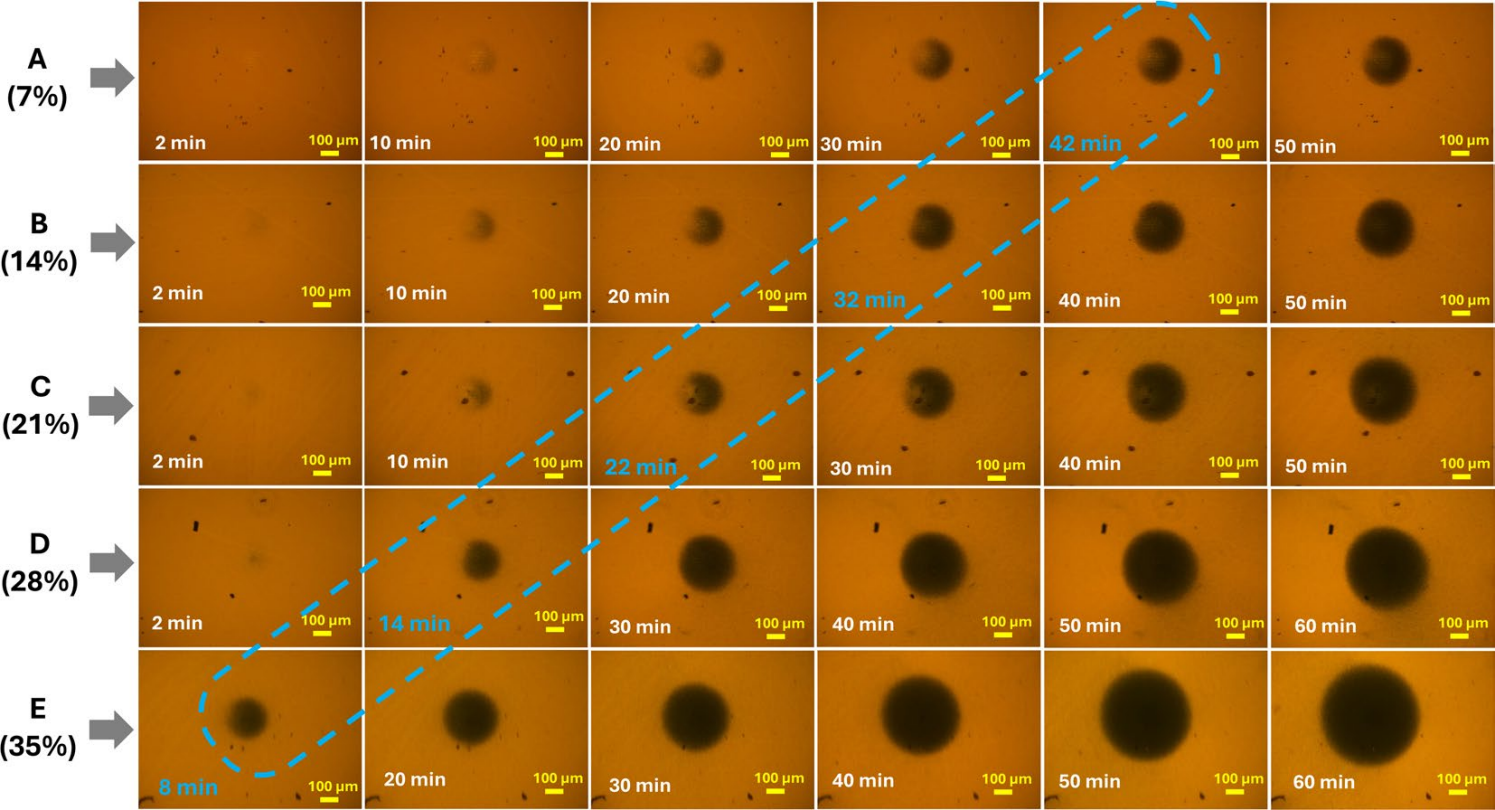
Microscopic images of the NP assemblies captured during SERS measurements for different NP densities (Magnification 10X). The assembly sizes at which optimum SERS signals were recorded are enclosed in a dotted oblong.

Microscopic images of the NP assemblies were captured during each SERS measurement to analyze the assembly size and density. Fig. 7 compares the captured images for the five NP densities, wherein images of the NP assembly at which the maximum SERS was observed are enclosed in an oblong. The average assembly radius for solutions A, B, C, D, and E at these points is estimated to be 117±5 μm, 124±2 μm, 119±3 μm, 118±6 μm, and 118±3 μm, respectively. These radius values for all the AuNP solutions of different AuNP densities exhibit minimal variation, with an average value of 119±1 μm. Thus, the optimum SERS signal for the colloidal solution of any NP density can be predicted just by monitoring the size of the NP assembly. This condition again brings us to the optimum nanogap situation for a better SERS signal and its relation to the assembly density. Below this assembly density, the interparticle nanogaps are wider, resulting in lower LSP hybridization between NPs, and above that, the nanogaps are narrower, triggering electron tunneling between the NPs, which leads to conduction and a drop in field localization. Moreover, at higher assembly density, the NP stacking in the z-direction leads to the masking of the SERS signal. Thus, the SERS intensity at 60 min for concentration A is higher because of the lower signal masking.

## Conclusion

In this study, we investigated the kinetics of SPP-assisted plasmonic assembly of Chi-AuNFs and its influence on the SERS intensity of analytes under study. The assembly kinetics is observed to be dependent on NP density due to varying NP flow rates for a given convection current of the fluid in the thermal gradient created by SPP heating. The SERS intensity of the analyte in the colloidal solution increases rapidly with assembly time and gradually decreases after peaking at different times for different NP densities. Notably, when the SERS intensity reaches the maximum, the measured assembly size remains constant for solutions of all NP densities. Moreover, the study revealed that the time taken to attain the optimum SERS intensity is inversely proportional to the NP density. Further, given that the NP concentration in the prepared colloidal solution varies for different synthesis protocols and successive attempts, it is essential to investigate the influence of NP density on the SERS.

## Supplementary Information

Below is the link to the electronic supplementary material.

Supplementary Material 1

## Data Availability

All the data pertaining to the study are already provided within the manuscript.
